# LncRNA expression in idiopathic achalasia: New insight and preliminary exploration into pathogenesis

**DOI:** 10.1515/med-2022-0473

**Published:** 2022-04-12

**Authors:** Chao Lu, Furong Wei, Xinjue He, Xin Yao, Chaohui Yu

**Affiliations:** Department of Gastroenterology, The First Affiliated Hospital, Zhejiang University School of Medicine, Hangzhou, 310003, China

**Keywords:** idiopathic achalasia, LES, microarray, lncRNA, pathogenesis, disorder

## Abstract

Idiopathic achalasia is a primary esophageal motility disorder characterized by the absence of esophageal peristalsis and impaired relaxation of the lower esophageal sphincter (LES). However, the pathogenesis of idiopathic achalasia remains unclear. To further understand the pathogenesis, we conducted lncRNA and mRNA microarray analyses. LES specimens from 5 patients and 4 controls were used for microarray. Potential target genes with significantly changed lncRNA and mRNA were predicted using cis/trans-regulatory algorithms, followed by the Gene Ontology and KEGG pathway enrichment analysis to understand the biophysical effect. Finally, 7,133 significantly dysregulated mRNAs (3,136 increased and 3,997 decreased), along with 6,892 significantly dysregulated lncRNAs (4,900 increased and 1,992 decreased). Biophysical function analysis revealed that the cell adhesion molecule (CAM) pathway was a common pathway. The predicted lncRNA targets of NRXN1 (Down FC: 9.07), NTNG2 (UP FC: 2.75), CADM1 (Down FC: 2.26), NLGN1 (Down FC: 4.60), NEGR1 (Down FC: 2.335), CD22 (Down FC: 5.62), HLA-DQB1 (Down FC: 5.06), and HLA-DOA (Down FC: 2.31) were inputted in this pathway, which was mainly located in the synapse part of the neural system and immune system. Our study demonstrates the lncRNAs and corresponding mRNAs that may play important roles in idiopathic achalasia.

## 1 Introduction

Idiopathic achalasia is a primary esophageal motility disorder characterized by the absence of esophageal peristalsis and impaired relaxation of the lower esophageal sphincter (LES) [1]. These abnormalities arise from the impairment of esophageal smooth muscle and the inhibitory neurotransmitter in LES [2]. The main clinical manifestations are dysphagia, regurgitation, esophageal retrosternal discomfort or pain, heartburn, and chronic pulmonary and bronchial lesions caused by reflux inhaled inducing symptoms of cough, sputum, dyspnea, and asthma [3]. Epidemiological data show that the annual incidence of idiopathic achalasia is relatively low, at approximately 1.6/100,000, with no gender specificity [4,5]. Although all age groups are susceptible, 30–60-year-old individuals have the highest incidence [4,5]. Boeckxstaens et al. have reported that in Iceland and Canada, the prevalence rate of idiopathic achalasia is relatively high, 8.7/100,000, and 10.8/100,000, respectively [6]. Although the incidence rates have been stable, the prevalence rates have increased with time [6]. This has increased interest and attention in idiopathic achalasia, as the exact etiology and pathogenesis remain unknown.

It is reported that the decrease in the number of myenteric neurons in LES caused by immunomediated inflammation is considered to be one of the possible causes of idiopathic achalasia [7]. The herpes simplex virus type 1 (HSV-1), measles virus, and human papillomavirus (HPV) are regarded as potential *in vivo* antigens. Activation of associated aberrant immune pathways, such as those involving cytotoxic T cell autoantibodies, via direct cytotoxicity or through HLA-mediated antigen recognition, has been thought to lead to the development of idiopathic achalasia [8,9,10]. Additionally, Boeckxstaens et al. have reported that a small number of idiopathic achalasia are associated with gene polymorphisms, such as HLA II molecules, vasoactive intestinal peptide receptor 1 (VIPR1), KIT, IL-10 promoter, and IL-23 receptor [6]. Palmieri et al. were the first to show that TLR4 and IL-18 were differentially expressed in idiopathic achalasia and that muscle nerve-related molecular pathways may exist in the event of an important role in disease development [11].

Here, we intended to compare and screen mRNA and long-chain non-coding RNA (lncRNA) of differentially expressed genes across the LES in the idiopathic achalasia and control groups using the gene chip technology to improve our understanding of the disease pathogenesis and identify novel clinical indicators for idiopathic achalasia.

## 2 Methods

### 2.1 Patients and specimens

LES specimens for idiopathic achalasia were obtained from 5 patients with idiopathic achalasia undergoing peroral endoscopic myotomy. A muscle slip measuring approximately 1 cm in length was taken from the proximal region of the LES. The control LES specimens were from 4 patients undergoing total gastrectomy for gastric cancer at the same time in our hospital. The average age of idiopathic achalasia patients was 46.4 ± 14.1 years, while the control was 65.3 ± 11.6 years. In addition, the average course of idiopathic achalasia was 76.80 ± 75.55 months. None of the patients received radiotherapy or chemotherapy before the operation. The tumor location was at least 2 cm away from the incisal margin of the esophagus, and subsequent pathology confirmed by an expert pathologist revealed that the tissues of the margins were determined to be LES, and there is no infiltration of cancer cells. Idiopathic achalasia was diagnosed according to radiological and endoscopic standards [12]. In addition, the test parameters of the sample met the quality control requirements, and the experiment was successful.


**Ethics approval and consent to participate:** The experimental protocols were approved by the Ethics Committee of Clinical Research, First Affiliated Hospital, College of Medicine, Zhejiang University (2017-545). All patients provided signed informed consent prior to participating in this study.

### 2.2 RNA isolation and lncRNA and mRNA microarray

The specimens were immediately stabilized in liquid nitrogen and stored at −80°C until RNA isolation. Total RNA was extracted using the TRIZOL Reagent (Cat#15596-018, Life Technologies, Carlsbad, CA, USA) and checked for RNA Integrity Number (RIN) to inspect RNA integrity using an Agilent Bioanalyzer 2100 (Agilent Technologies, Santa Clara, CA, US). Qualified total RNA was further purified using an RNeasy mini kit (Cat#74106, QIAGEN, GmBH, Germany) and RNase-Free DNase Set (Cat#79254, QIAGEN, GmBH, Germany). The purity and concentration of RNA were determined from OD260/280 readings using a spectrophotometer (NanoDrop ND-1000). RNA integrity was determined by 1% formaldehyde denaturing gel electrophoresis.

Total RNA was amplified and labeled using the Low Input Quick Amp Labeling Kit, One-Color (Cat#5190-2305, Agilent Technologies, Santa Clara, CA, USA). Labeled cRNA was purified using Nucleospin^®^ RNA clean-up (Cat#740.948.250, MACHEREY-NAGEL, Germany). Each slide was hybridized with 1.65 μg of Cy3-labeled cRNA using the Gene Expression Hybridization Kit (Cat#5188-5242, Agilent Technologies, Santa Clara, CA, USA) in a Hybridization Oven (Cat#G2545A, Agilent Technologies, Santa Clara, CA, USA). After hybridization, slides were washed in staining dishes (Cat#121, Thermo Shandon, Waltham, MA, US) with the Gene Expression Wash Buffer Kit (Cat#5188-5327, Agilent Technologies, Santa Clara, CA, USA).

The chip was scanned with an Agilent chip scanner (Agilent Technologies, Santa Clara, CA, USA). The Agilent Feature Extraction software 10.7 (Agilent Technologies, Santa Clara, CA, USA) was used to analyze and extract data from the hybrid images. Data were normalized using the Quantile algorithm, and the differences in gene expression were analyzed using the Agilent GeneSpring software 11.0 (Agilent Technologies, Santa Clara, CA, USA).

### 2.3 Bioinformatics and statistical analyses

Target gene prediction was divided into cis-/trans-prediction. Predicting cis-/trans-regulatory effects of the reported algorithms was used to identify target genes of lncRNA [13]. The genes transcribed within a 10-kbp window upstream or downstream of lncRNA were considered cis target genes [14]. The trans-analysis was tested using RNAplex v0.2, which is a fast tool for RNA-RNA interaction searches that neglects the intramolecular interactions and uses a simplified energy model [15]. KOBAS (KEGG Orthology Based Annotation System) was used to analyze the GO (Gene Ontology) functional annotation and the KEGG (Kyoto Encyclopedia of Genes and Genomes) biological pathways of different mRNA and lncRNA target genes [16].

Log FC, which was the criterion of statistical significance, was defined as the ratio of the standard values of the hybridization signal between the experimental and para-carcinoma tissue. The conventional screening criteria for different genes were as follows: according to the previous reports, the FC value was more than twice when statistically different [17]. The greater the difference in FC value, the greater the difference between the two samples. Additionally, the smaller the *P*-value, the higher the reliability of the difference among genes. Statistical significance was set at *P* < 0.05.

### 2.4 Construction of the coding-non-coding gene co-expression network

According to the correlation analysis of the differential expressed lncRNA and mRNA, the coding-non-coding gene co-expression network (CNC network) was constructed. For each pair of genes, the Pearson correlation was calculated, and the significant correlation pairs were chosen to construct the network. The network would be drawn through the open-source bioinformatics software Cytoscape based on lncRNAs and mRNAs with Pearson correlation coefficients of more than 0.99. In network analysis, degree of centrality is defined as the link numbers one node has to the other. A degree is the simplest and most important measure of a gene centrality within a network that determines the relative importance [18].

### 2.5 Cis-acting lncRNA prediction

The cis-acting lncRNA prediction was performed by their tight correlation to a group of expressed protein-coding genes. The lncRNA resided at genomic loci where a protein-coding gene and a lncRNA gene are located within 10 kb of each other along the genome [19]. Therefore, “cis” refers to the same locus regulatory mechanisms, which include antisense-mediated regulation by lncRNAs of protein-coding genes that are encoded in the same locus.

### 2.6 Trans-acting lncRNA prediction

The trans-prediction was conducted using blat tools (Standalone BLAT v. 35 × 1 fast sequence search command-line tool) to compare the full sequence of the lncRNA with the 3′UTR of its co-expression mRNAs, with the default parameter setting.

## 3 Results

### 3.1 Clinical characteristics

A total of five patients with idiopathic achalasia were included in the chip analysis, including two males and three females. The average age of patients was 46.4 ± 14.1 years, and the average course was 76.80 ± 75.55 months. Major clinical manifestations of patients included dysphagia (100%), reflux (100%), respiratory reactions (20%), chest pain (40%), heartburn (20%), and weight loss (80%). However, the symptoms of the control were mainly weight loss (100%) and upper abdominal pain (75%), and there were no reflux, heartburn, or dysphagia. The average Eckardt score was 6.4 ± 1.3 (Table 1).

**Table 1:** Clinical characteristics of patients and controls

	Study group	Control group
Age	46.4 ± 14.1	65.3 ± 11.6
Gender (male/female)	2/3	2/2
Course (month)	76.80 ± 75.55	—
Eckardt score	6.4 ± 1.3	—
Symptoms		
Dysphagia	5	0
Reflux	5	0
Respiratory reaction	1	0
Chest pain	2	0
Heartburn	1	0
Weight loss	4	4
Upper abdominal pain	0	3

### 3.2 Microarray gene expression analysis

**Figure 1:**
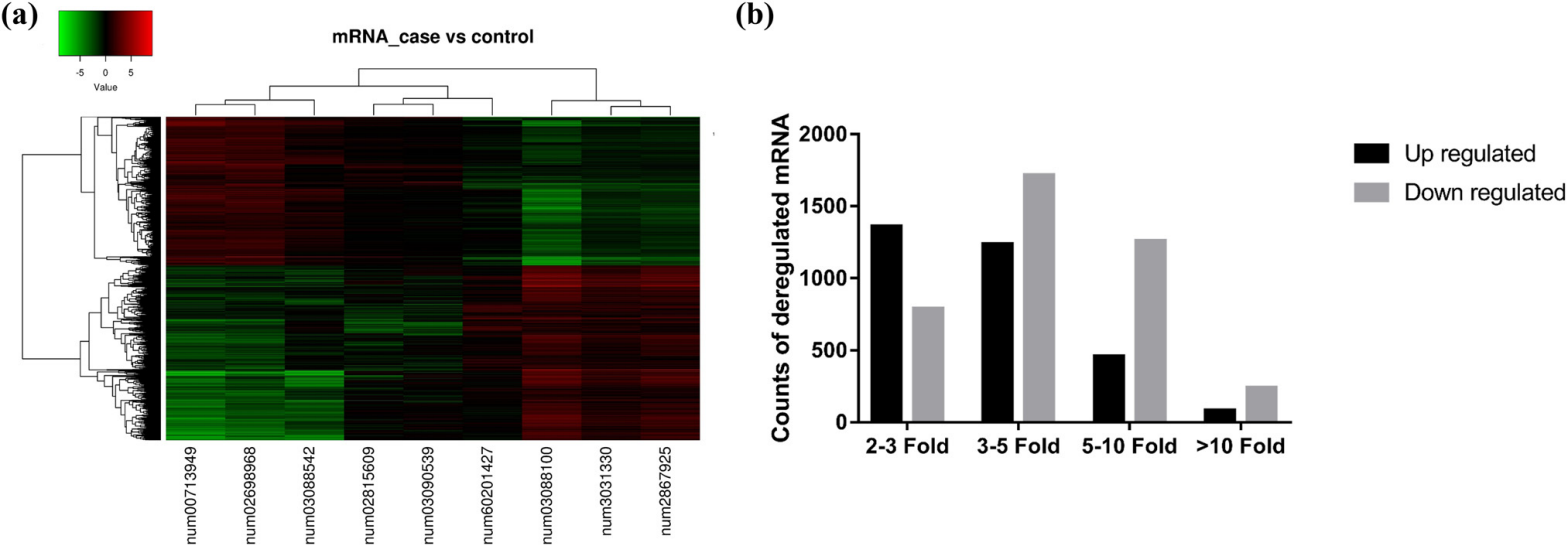
Microarray results of mRNA profile in idiopathic achalasia. (a) Heat map of differentially expressed mRNAs in idiopathic achalasia. Each row shows relative levels of expression for a single mRNA, and each column represents the expression levels for a single sample. Colors indicate relatively high or low expression, respectively (red is high expression and green is a low expression). (b) Expression profiling of mRNAs in idiopathic achalasia and control. The determination of upregulated and downregulated mRNAs was based on more than two-fold changes in the normalized probe signal intensity in idiopathic achalasia compared to that in the control. Black columns represent the number of upregulated mRNAs, whereas the gray columns represent the number of downregulated mRNAs.

**Figure 2:**
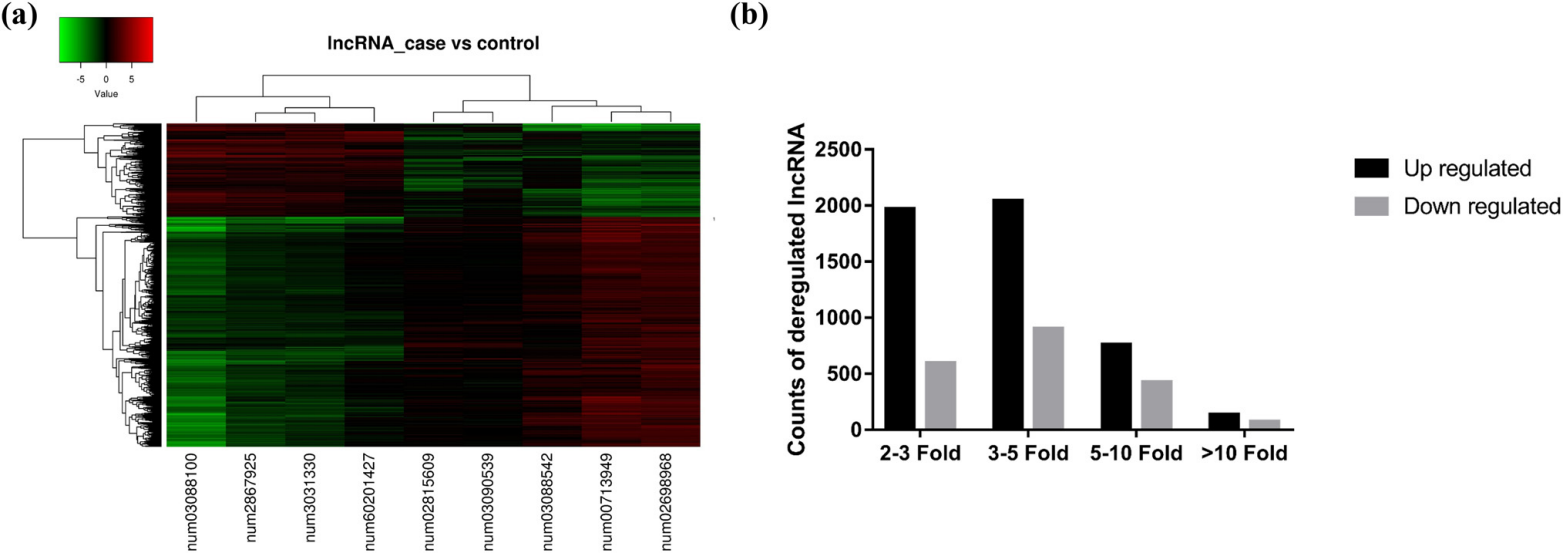
Microarray results of lncRNA profile in idiopathic achalasia. (a) Heat map of differentially expressed lncRNAs in idiopathic achalasia. Each row shows relative levels of expression for a single lncRNA, and each column represents the expression levels for a single sample. Colors indicate relatively high or low expression, respectively (red is a high expression and green is a low expression). (b) Expression profiling of lncRNAs in idiopathic achalasia and controls. The determination of upregulated and downregulated lncRNAs was based on more than two-fold changes in the normalized probe signal intensity in idiopathic achalasia compared to that in the control. Black columns represent the number of upregulated lncRNAs, whereas the gray columns represent the number of downregulated lncRNAs.

By comparison, microarray analysis revealed a total of 14,025 differentially expressed genes, including 7,133 differentially expressed mRNA (3,136 upregulated and 3,997 downregulated) (Figure 1) and 6,892 lncRNA (4,900 up-regulated and 1,992 down-regulated) (Figure 2).

Among the 3,136 elevated mRNAs, expression of 83 mRNAs increased considerably by more than 10-fold, expression of 459 mRNAs increased moderately by 5- to 10-fold, expression of 1,235 mRNAs increased mildly by 3- to 5-fold, and expression of 1,359 mRNAs increased slightly by a 2- to 3-fold change (Figure 1b). Of the 3,997 decreased mRNAs, expression of 239 mRNAs increased considerably by more than 10-fold, expression of 1,255 mRNAs increased moderately by 5- to 10-fold, expression of 1,714 mRNAs increased mildly by 3- to 5-fold, and expression of 789 mRNAs increased slightly by a 2- to 3-fold change (Figure 1b).

Of the 4,900 elevated lncRNAs, expression of 135 lncRNAs increased considerably by more than 10-fold, expression of 758 mRNAs increased moderately by 5- to 10-fold, expression of 2,040 mRNAs increased mildly by 3- to 5-fold, and expression of 1,967 mRNAs increased slightly by a 2- to 3-fold change (Figure 2b). Additionally, expression of 1,992 decreased lncRNAs, including 73 lncRNAs, increased considerably by more than 10-fold, expression of 425 mRNAs increased moderately by 5- to 10-fold, expression of 902 mRNAs increased mildly by 3- to 5-fold, and expression of 593 mRNAs increased slightly by a 2- to 3-fold change (Figure 2b).

The most upregulated and downregulated genes are reported in Tables 2 and 3. mRNAs in patients included LMO7 (FC + 26.7, *P* = 6.03^−5^), ITGA8 (FC − 55.6, *P* = 2.58^−3^), RPS4Y2 (FC – 47.8, *P* = 1.10^−3^), and so on. LncRNAs included TCONS_0001118 (FC + 72.6, *P* = 3.01^−5^), ENST00000447880.1 (FC + 45.1, *P* = 4.72^−5^), ENST00000457658.1 (FC – 30.9, *P* = 8.91^−5^), ASO3473 (FC – 28.3, *P* = 1.45^−4^), and so on.

**Table 2:** The most up-regulated and down-regulated mRNA

Gene symbol	*P*	FC (abs)
LMO7	6.03318 × 10^−5^	26.71425695
TMEM200C	3.28108 × 10^−5^	25.94489757
C22orf39	3.66968 × 10^−5^	25.87756057
LHX5	5.35747 × 10^−6^	24.72500133
CRNN	0.027830519	24.03878997
SPRR3	0.017117696	23.78862026
WI2-2373I1.2	0.000100648	23.66951699
ENST00000442466	0.001293632	22.33677558
TBR1	7.6705 × 10^−5^	21.22063752
AAK1	7.93936 × 10^−5^	19.70644678
ITGA8	0.002581062	−55.60137848
RPS4Y2	0.001103543	−47.82133021
BRWD3	0.023419339	−31.10210048
FLRT2	0.000220524	−30.55600013
FOXP2	0.013681404	−29.06785335
SCARA5	0.000218602	−28.83943377
PTAR1	0.026496723	−27.49359787
P2RY14	0.000304615	−26.79329943
SLC31A1	0.001766187	−26.33664678
DDX3Y	4.30241 × 10^−5^	−26.26270772

**Table 3:** The most up-regulated and down-regulated lncRNA

lncRNA ID	*P*	FC (abs)
TCONS_00011185	3.01402 × 10^−5^	72.64201265
ENST00000447880.1	4.71544 × 10^−5^	45.08069048
RNA147445|p0549_imsncRNA190	8.44416 × 10^−5^	33.92913857
ENST00000512637.1	0.000197358	32.89494311
XR_158741.1	3.81046E × 10^−6^	32.63342097
XR_245991.1	1.32945 × 10^−5^	31.70148851
ENST00000507924.1	2.82601 × 10^−5^	29.59564156
ENST00000605571.1	0.000329898	29.01344488
ENST00000511301.1	3.32355 × 10^−5^	28.39455549
XR_110047.1	2.72823 × 10^−5^	27.21027354
ENST00000457658.1	8.91147 × 10^−5^	−30.89314361
ASO3473	0.000145592	−28.27190349
TCONS_00029197	3.81342 × 10^−6^	−25.04219798
ENST00000582940.1	0.008796621	−24.34463314
TCONS_00017647	0.003232599	−23.52995468
ENST00000422971.1	0.001210922	−21.38524635
XR_427728.1	0.001271232	−20.83603776
ENST00000604219.1	0.011846474	−19.2863216
ENST00000434656.1	0.006403102	−17.25903289
uc.480-	0.010963956	−16.26472502

### 3.3 GO and KEGG pathway analyses of differentially expressed genes

**Figure 3:**
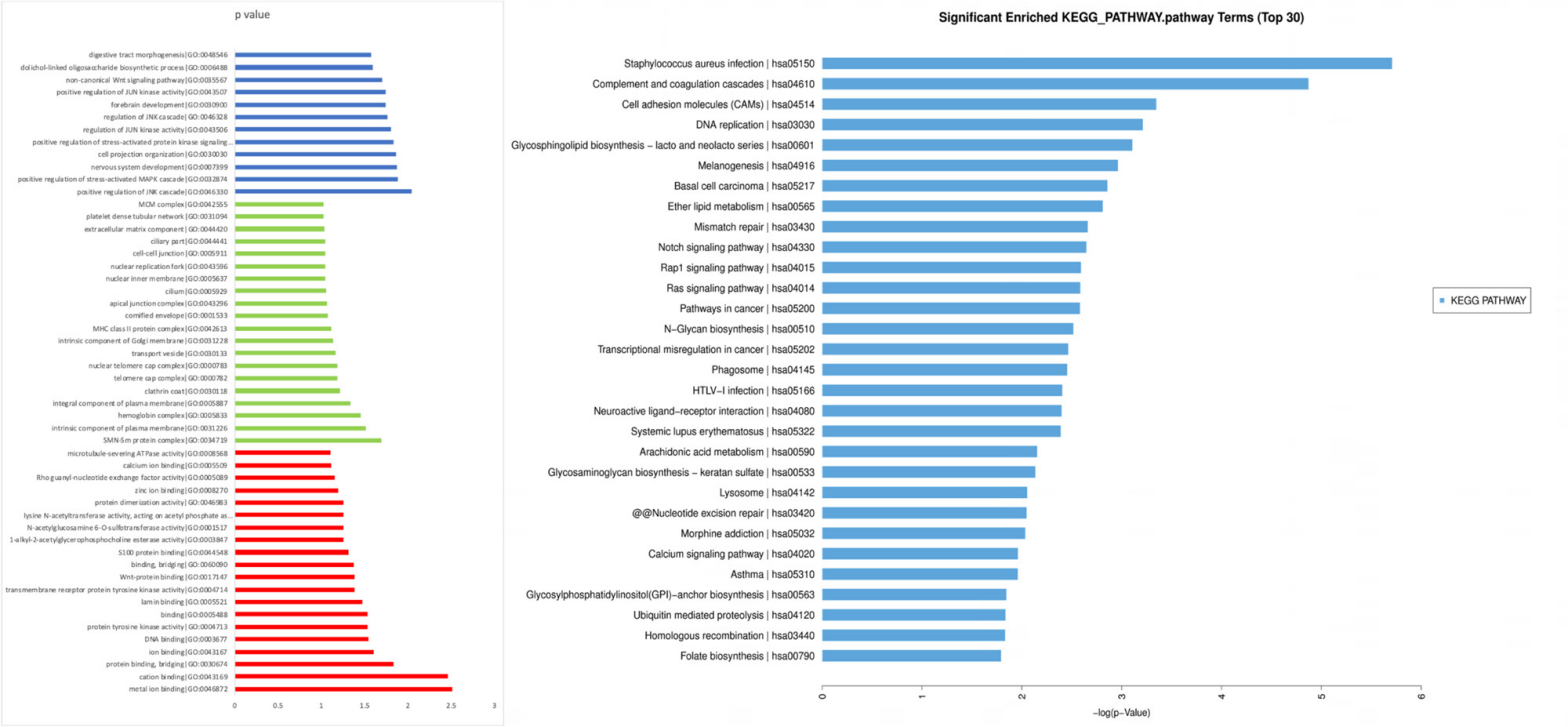
GO analysis of the predicted mRNA target genes. Data are presented as a histogram of the relevant biological processes, cellular components, and molecular functions identified. KEGG pathway analysis of predicted mRNA is presented as a potential pathway.

To help interpret the biological functions of altered mRNA and lncRNA profiles, GO and KEGG pathway analyses were performed. Among the different mRNA biological processes, GO analysis showed anatomical structure morphogenesis, positive regulation of JNK cascade, positive regulation of stress-activated MAPK cascade, nervous system development, cell projection organization, and fatty acid derivative biosynthetic processes might be related to the disease (Figure 3). To gain further insights into the pathogenesis of idiopathic achalasia, signaling pathway enrichment analysis was performed using the KEGG database. The following pathways showed significant transcriptional changes: *Staphylococcus aureus* infection (*P* = 0.0033), complement and coagulation cascades (*P* = 0.0076), cell adhesion molecules (CAMs) (*P* = 0.0351), DNA replication (*P* = 0.0402), and glycosphingolipid biosynthesis – lacto and neolacto series (*P* = 0.0446) (Figure 3).

**Figure 4:**
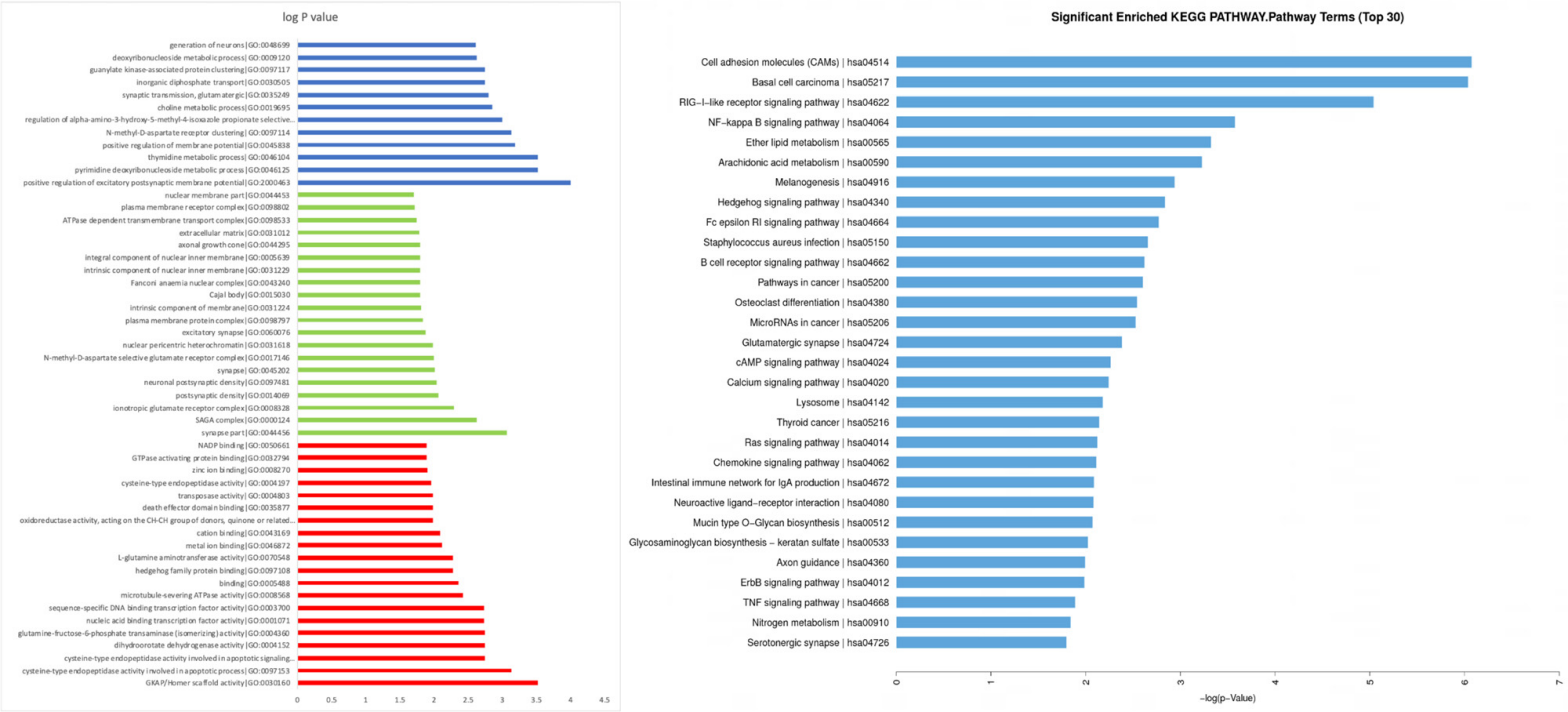
GO analysis of the predicted lncRNA target genes. Data are presented as a histogram of the relevant biological processes, cellular components, and molecular functions identified. KEGG pathway analysis of predicted lncRNAs is presented as a potential pathway.

GO analysis of differentially expressed lncRNAs revealed that the following biological processes were altered in idiopathic achalasia: positive regulation of excitatory postsynaptic membrane potential, pyrimidine deoxyribonucleoside metabolic process, and positive regulation of membrane potential (Figure 4). Significant KEGG analysis changes in lncRNAs were identified as CAMs (*P* = 0.0023), basal cell carcinoma (*P* = 0.0024), RIG-I-like receptor signaling pathway (*P* = 0.0065), NF-kappa B signaling pathway (*P* = 0.0279), ether lipid metabolism (*P* = 0.0360), and arachidonic acid metabolism (*P* = 0.0396) (Figure 4).

### 3.4 Common pathways of differentially expressed lncRNAs and corresponding mRNAs

Analyses of the functions of the differentially expressed mRNA and lncRNA target genes revealed that the CAM pathway was a common pathway. The mRNA pathway analysis found that 55 genes were inputted into the CAM pathway for annotation along with 142 background genes. The total input and background genes were 6,783 and 23,740, respectively. In the lncRNA target gene pathway analysis, the number of background genes annotated into the CAM pathway was 142 and the number of input genes was 14; CLDN11 (Down FC: 3.42), NRXN1 (Down FC: 9.07), NCAM2 (UP FC: 3.11), HLA-DQB1 (Down FC: 5.06), NTNG2 (UP FC: 2.75), HLA-DOA (Down FC: 2.31), CADM1 (Down FC: 2.26), CLD-N18 (Down FC: 3.94), CD22 (Down FC: 5.62), ITGA6 (Down FC: 10.43), NEGR1 (Down FC: 2.335), MPZL1 (Down FC: 4.31), CNTNAP2 (Down FC: 5.88), and NLGN1 (Down FC: 4.60).

## 4 Discussion

Although the incidence of idiopathic achalasia continues to increase, its pathogenesis remains unknown. Owing to various conditions such as the difficulty of animal experimentation and the limitations of in vitro studies, disease pathogenesis determination has traditionally been difficult. Some reports suggest a correlation between the onset of idiopathic achalasia and the destruction of myenteric neurons and inflammatory response [20,21,22]. We focused on the lncRNA expression spectrum in patients with idiopathic achalasia, together with mRNA, to elucidate the molecular mechanisms involved in disease pathogenesis. To further characterize changes in lncRNA and mRNA profiles, GO and pathway analyses were conducted. Among all, the CAM pathway was commonly identified.

The muscle specimen from POEM and gastrectomies were both circular muscles. For surgical specimens, we would ask the surgery to isolate the circular muscle, and then invite a pathologist to identify the specimens. For POEM specimens, due to the small amount of specimen tissue, we did not conduct histological identification of achalasia samples. However, based on the direct observation of the operation and the comprehensive analysis of the patient’s prognosis after the circular muscle segment during the operation, it is certain that the specimen is circular muscle. After tissue retrieval, we immediately saved it in liquid nitrogen and then performed RNA extraction within 24 hours.

Analysis of the 14 target genes in the CAM pathway revealed that they were mainly concentrated in the synaptic region (e.g. NRXN1, NTNG2, CADM1, NLGN1, NEGR1) and immune system (CD22, HLA-DQB1, HLA-DOA). NRXN1, which plays an important role in the formation, maturation, and neurotransmission of synapses, is expressed in the presynaptic membrane and interacts with some adhesion molecules in the postsynaptic membrane [23]. NRXN1 has not been reported to be related to idiopathic achalasia. Zeng et al. have revealed that NRXN1 deletions affect several biological processes during neurodevelopment, including synaptic adhesion and neuron differentiation, based on *in vitro* models [24]. In our study, there was a significant down-regulation of NRXN1 (Down FC: 9.07), suggesting that NRXN1 might be related to the onset of idiopathic achalasia. However, whether the downregulation of NRXN1 induces the inhibitory effect of the LES myenteric plexus requires further study.

Netrin-G2 (NTNG2) is a nerve axon-directing factor expressed mainly in the central nervous system and plays a role in the formation of axons and the migration of nerve cells [25]. If there is abnormal synaptic remodeling caused by the corresponding upregulation of NTNG2 expression in the LES, there may be evidence that high expression of NTNG2 is associated with idiopathic achalasia. Pan et al. have reported that high expression of NTNG2 in the pathophysiology of epilepsy may participate in the abnormal development of synapses and neuronal migration [26].

Cell adhesion molecule 1 (CADM1) is mainly involved in calcium-independent cell-to-cell adhesion and signal transduction, whose low expression is associated with some tumors. Ito et al. have reported that CADM1 can also promote the connection between neurons and mast cells, mast cells, and smooth muscle, suggesting that it may play an important role in the neural-immune pathway [27]. Additionally, Frei et al. have reported that CADM1 plays an important role in the formation of synapses and early neural pathways and that downregulation of CADM1 interferes with selective axon–axon interactions, leading to abnormal axon pathfinding [28]. These results combined with chip results suggested that CADM1 might participate in the pathogenesis of idiopathic achalasia through neuro-immune pathways and affect the normal morphology and function of synapses.

Neuroligin-1 (NLGN1), a member of the NLGN gene family, is located in the postsynapse, which can directly interact with *N*-methyl-d-aspartate (NMDA) receptors by binding to postsynaptic density protein-95 (PSD95), and then participates in the formation and functional regulation of excitatory synapses [29]. In the enteric nervous system (ENS) of rats, researchers found that NLGN1 was expressed and had a temporal correlation with the development of ENS, during which malformations might occur due to their disruptions [30]. Zhang et al. found that the expression of NLGN1 decreased significantly in the intestinal stenosis in Hirschsprung Disease (HSCR) [31]. Decreased expression of NLGN1 may cause abnormal excitatory synaptic conduction, leading to continued contraction of diseased intestinal segments, and eventually the onset of HSCR. Histologically, it has been confirmed that ganglion cells in the esophageal smooth muscle myenteric plexus are reduced in idiopathic achalasia. Whether the down-regulation of NLGN1 causes synapse formation and information transmission in the LES myenteric plexus, which is also the enteric nervous system, and then affects the peristaltic function of the esophagus, warrants further study.

A cluster of differentiation 22 (CD22) is a cell surface adhesion factor mainly expressed in mature B cells, which regulates B cell activation and helps control the sensitivity of antigenic responses [32]. CD22 negatively regulates BCR activation, thereby inhibiting B cell signaling and affecting B cell proliferation, differentiation, and homeostasis. Mutation or abnormal expression of CD22 causes autoimmune disorders through the BCR signaling pathway [33]. No related article was reported regarding CD22 and idiopathic achalasia. In this study, the chip results showed that CD22 expression was significantly downregulated (Down FC: 5.62). Abnormal BCR signaling pathways may be caused by the downregulation of CD22 expression in the LES as well as the inflammatory response to its own intermuscular neurons, which leads to the weakening of the diastolic response and the sustained high pressure of the muscle.

HLA-DQB1 has been reported to be associated with idiopathic achalasia. Gockel et al. have reported that mutations in certain loci of HLA-DQB1 increase the risk of idiopathic achalasia by screening large samples [21]. A previous study on white subjects revealed a significant association between idiopathic achalasia and the DQB1*0602 allele [34]. Our findings validate the previous study and indirectly demonstrate the reliability of the chip results.

For the first time, we have discovered the possibility of the CAM pathway regulating the occurrence and development of idiopathic achalasia and derived some differentially expressed genes. The scope and limitations of our study are discussed below. We selected patients undergoing total gastrectomy for gastric cancer as the control group. If we select the esophageal muscle tissues of patients with idiopathic achalasia away from the LES as a secondary control, tissue comparison of different parts of the same patient will generate more reliable results. Additionally, further studies are needed to investigate the molecular mechanisms related to CAM participation in the pathogenesis of idiopathic achalasia. In addition, this study intended to conduct stratified analysis according to factors such as age, gender, course, and Eckardt score, to further explore the influence of different factors on gene expression. However, based on the limitation of samples, we have not been able to complete a stratified analysis. We were well aware of the limitations of results imposed by the lack of sample, and we are actively expanding the sample to make the results more reliable and to verify differentially expressed genes.

In summary, our data provide a rigorously characterized expression profile of the lncRNA and mRNA in the LES of patients with idiopathic achalasia. A total of 3,136 significantly upregulated and 3,997 significantly downregulated mRNAs and 6,892 significantly dysregulated lncRNAs (4,900 increased and 1,992 decreased) were identified. Particularly, we are the first to pinpoint the role of CAMs as a major pathway involved in the development of idiopathic achalasia. The current study provides new insights into the pathogenesis of idiopathic achalasia involving lncRNAs. Further studies should focus on the molecular mechanisms impacted by these changes.
